# A Study on the Biodiversity of Pigmented Andean Potatoes: Nutritional Profile and Phenolic Composition

**DOI:** 10.3390/molecules25143169

**Published:** 2020-07-10

**Authors:** Maria Bellumori, Nancy A. Chasquibol Silva, Laida Vilca, Luisa Andrenelli, Lorenzo Cecchi, Marzia Innocenti, Diletta Balli, Nadia Mulinacci

**Affiliations:** 1 Department of NEUROFARBA, University of Florence, Nutraceutical and Pharmaceutical section, via U. Schiff 6, Sesto F.no, 50019 Florence, Italy; maria.bellumori@unifi.it (M.B.); lo.cecchi@unifi.it (L.C.); marzia.innocenti@unifi.it (M.I.); diletta.balli@unifi.it (D.B.); 2 Center of Studies and Innovation of Functional Foods (CEIAF), Faculty of Industrial Engineering, Institute of Scientific Research, IDIC, University of Lima, Avda. Javier Prado Este, 4600 Surco, Lima 33, Peru; Nchasquibol@ulima.edu.pe (N.A.C.S.); Lvilca@ulima.edu.pe (L.V.); 3 Department of Agriculture, Food, Environment and Forestry University of Florence, Piazzale delle Cascine 18, 50144 Florence, Italy; luisa.andrenelli@unifi.it

**Keywords:** phenolic compounds, anthocyanins, neochlorogenic acid, minerals, proximate composition, HPLC–DAD–MS analysis

## Abstract

The characterization of six varieties of native Andean potatoes with a wide biodiversity in tuber shape, flesh, and skin color was performed, through the determination of their proximate composition, mineral content, and phenolic profile. Minerals concentration revealed significant genotypic variation. Potassium was the most abundant element in all varieties, ranging from 7272.9 to 13,059.9 µg/g and from 12,418 to 17,388.6 µg/g dried weight for the flesh and skin samples, respectively. Iron content was relevant, ranging from 20.5 to 39.9 µg/g and from 112.2 to 288.8 µg/g dried weight in flesh and skin samples, respectively. Phenolic compounds were consistently higher in the skin than in the flesh. The total content varied greatly from 19.5 to 2015.3 µg/g and from 1592.3 to 14807.3 µg/g dried weight for flesh and skin tissues, respectively. 5-caffeoylquinic acid was 74% of the total phenolic acids. Different pattern of anthocyanins was found, depending on the color of the variety; the red genotypes contained predominantly pelargonidin derivatives, while the purple samples had petunidin as a major anthocyanidin. This study increases the knowledge of the composition of the local Andean varieties (which are only scarcely studied so far), helping to enhance these genotypes and the conservation of biodiversity.

Academic Editor: Pierluigi Plastina

## 1. Introduction

Potato domestication began in 7000 BC and the Andean region of Peru has so far preserved a rich biodiversity. This tuber is a typical product of the Andean culture that has conserved its resources for generations, and it is currently recognized as an important legacy for humanity. Different shapes, colors, textures and flavors of potatoes have been maintained and appreciated by rural families for centuries, using the tuber in the preparation of soups, stews, and traditional dishes like “pachamanca”, prepared as reported in the oldest traditional culinary technique in Peru.

Peru is the largest producer of potatoes in Latin America and the twelfth in the world, with 4.5 million tons per year. Here, the potato has a sort of transcendental importance in the social and economic aspect and in fact 730,000 families depend on its cultivation [1].

Many studies highlighted the importance of investigating the characterization of local varieties and promoting the conservation and the recovery of local biodiversity [2,3,4].

Andean genotypes show the widest biodiversity in tuber shape, flesh and skin color, texture, and flavor, and this might lead to a considerable variability in nutritional and bioactive compound contents, as highlighted in several studies [5,6,7,8].

Compared to white and yellow commercial potatoes, red- and purple-fleshed and skinned varieties contain significantly higher amounts of antioxidant phenolic compounds. Particularly, both classes of anthocyanins and their biosynthetic precursors, the cinnamic acids, are in higher concentration in these varieties [9,10,11]. The potato genotype is the most determining factor that affect the profile and the content of phenolic compounds, besides the environmental conditions that can influence the quantity of these molecules in the tuber [6]. Modern agricultural practices and climate change are contributing to the loss of potato biodiversity, and thus, the loss of the genes coding for nutrient biosynthetic pathways [3].

In order to preserve the crop genetic diversity and support regional producers in maintaining the diversification of their production to avoid the biodiversity loss, it is extremely important to study the characteristics of local Andean potatoes.

The aim of this study was to deepen the knowledge about Andean potatoes, by studying the chemical composition of six native pigmented varieties, which are among the staple food crops of the Huancavelica region of Peru—Puma Makin, Leona, Yawar Manto, Añil, Sangre de Toro, and Qequrani. For this purpose, several analyses were carried out on flesh and skin tissues: (i) the proximate analysis and the determination of macro- and microelements was done by ICP–OES iCAP, (ii) the determination of phenolic acids and anthocyanins was done by HPLC–DAD–MS, and (iii) the evaluation of carotenoid content in the flesh tissue was done by HPLC–DAD.

Greater knowledge of the nutritional and functional properties of Andean varieties can contribute to the conservation of biodiversity of Peru’s regional heritage. As far as we know, no data are available so far on the composition of these pigmented varieties.

## 2. Results and Discussion

### 2.1. Morphological Aspects and Proximate Composition

**Figure 1:**
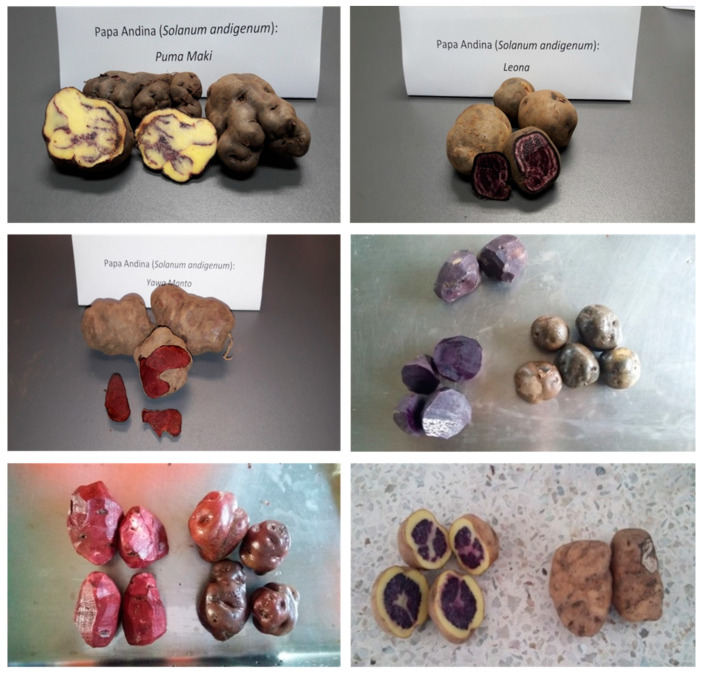
Varieties of Andean potatoes—Puma Makin, Leona (first line), Yawar Manto, Añil (second line), and Sangre de Toro, Qequrani (third line).

The different morphological characteristics of the six Andean varieties of potatoes mainly with regards to their color, size, and shape are summarized in Figure 1 and Table 1. Table 2 shows the proximate composition of the different varieties evaluated separately for their flesh and skin.

As expected, energy was derived mostly from carbohydrates. A wide range of carbohydrates content (from 18.0 to 34.1 g/100 g on fresh weight) was found in the flesh samples, corresponding to energy values of 79.7– 90.6% Kcal/100 g. The same trend was observed for the carbohydrates in the skin samples (from 18.3 to 33.3 g/100 g) with a contribution of 80.1–88.7% Kcal/100 g. These results were in some cases higher than those reported in the literature. Jimenez et al [12] studied 7 varieties of Andean potatoes and reported a carbohydrates content ranging from 12.6 to 22.6 g/100 g fresh weight; analogously, Calliope et al. [8] showed values from 11.9 to 24.0 g/100 g, analyzing 44 different genotypes of Andean potatoes. According to these and other authors [2,8], the observed variability might be due to genotype, reported as one of the most significant factors affecting the nutritional characteristics of different crops.

Moisture content ranged from 61.4 to 76.5 g/100 g in flesh samples and from 61.9 to 76.8 g/100 g in skin samples, according to Burlingame et al. [3], who observed similar values in 51 varieties of different origin. Fat content was always below the 1% and varied from 0.1 to 0.7 g/100 g in flesh samples, and from 0.1 to 0.6 g/100 g in skin samples. Protein content showed a certain variability with a range from 1.3 to 3.6 g/100 g in flesh and from 2.7 to 4.4 g/100 g in skin, similar to what was observed by other authors [3,8]. Ash content ranged from 1.2 to 1.7 g/100 g in flesh and from 1.0 to 1.4 g/100 g in skin, with similar values reported by Jimenez et al. [12].

### 2.2. Mineral Content

Potato flesh and skin are interesting sources of both macro and microelements, mostly potassium, phosphorus, magnesium, calcium, and iron. Mineral concentrations in tubers were highly variable among the six genotypes (Table 3) and statistically significant differences (*p* < 0.05) were observed. Overall, the contents of the elements are in the range of those determined by other authors [5,13].

As previously reported, potatoes are known as an excellent source of potassium [14,15]. This element was the most abundant in all the studied varieties and its content in the flesh varied from 7272.9 ± 129.7 to 13059.9 ± 36.5 μg/g of dry weight (DW), while it ranged from 12,418 ± 209.6 to 17388.6 ± 211.1 μg/g DW for the skin samples. The Yawar Manto variety showed the highest concentration of this macroelement, in both flesh and skin samples, while the Añil variety showed a similar value only in the skin tissue, not highlighting significant differences compared to the Yawar Manto variety.

As reported in Table 3, all elements showed higher concentrations in the skin samples than those in the flesh samples for all analyzed genotypes, in agreement with the results reported by other authors [16]. The Yawar Manto variety showed the highest amount of phosphorus among the flesh samples (1252.1 ± 21.1 μg/g DW), while the Leona variety showed the highest content (2124.9 ± 24.9 μg/g DW) in the skin samples. The phosphorus amount was significantly lower in the Sangre de Toro variety, both in the flesh and in the skin samples. The magnesium content in the flesh samples varied from 312.1 ± 3.8 to 591.4 ± 8.7 μg/g for the Sangre de Toro e Yawar Manto varieties, respectively; for the skin samples, the Sangre de Toro variety again showed the lowest amount (414.2 ± 13.7 μg/g), while this element was more abundant in the Añil variety (833.1 ± 12.8 μg/g).

Calcium is an essential mineral involved in neuromuscular function, blood clotting and many metabolic processes [5], with a daily intake often below the recommended dose. Among the samples, the Sangre de Toro variety showed the highest content in calcium, with values of 361.9 ± 7.8 and 953.5 ± 10.1 μg/g DW in the flesh and in the skin, respectively; no statistically significant differences were observed for the skin tissue between this variety and the Qequrani, which showed a value of 943.6 ± 7.9 μg/g DW. On the other hand, the Añil variety showed the lowest content in both flesh and skin samples, with levels of 126.2 ± 13.4 and 443.4 ± 5.1 μg/g DW, respectively. As reported in Table 3, this element showed a large difference between the two tissues; the values reported for the skin were approximately three times higher than those in the flesh samples, for all potato genotypes.

According to a recent survey in Peru, women and children in the Department of Huancavelica, a region with one of the country’s highest rates of poverty and malnutrition, use to consume on average 800 g and 200 g, respectively, of potatoes per day [17]. The FAO’s Recommended Nutrient Intake (RNI) for calcium depends on the bioavailability of the mineral; assuming a bioavailability of around 40%, the RNI for calcium is 1000 mg/day for adults and 400 mg/day for children. Taking into account the moisture content, the highest calcium-ranking variety (Sangre de Toro, Table 3) contained 120.2 and 363.3 μg/g of fresh weight (FW) of calcium in the flesh and in the skin tissues, respectively. Considering the consumption of the whole tuber, a mean value of calcium concentration of 79.4 g/100 g was calculated, taking into account the average flesh-to-skin ratio registered by Jimenez et al. [12], for seven Andean potato varieties. Thus, the Sangre de Toro genotype (mean calcium concentration of 132.8 μg/g FW) could provide, on average, 10.6% of the daily dietary calcium intake and 6.6% for women and children, respectively.

Microelements such as iron and zinc are essential for a good health maintenance, and potato flesh and skin represent an interesting source, as highlighted by Andre et al. [5] and Burgos et al. [17]. The iron content in the six potato genotypes varied from 20.5 ± 1.2 to 39.9 ± 6.5 μg/g DW in the flesh tissue and from 112.2 ± 6.2 to 288.8 ± 5.3 in the skin samples. These values were higher than those reported in the literature by Andre et al. [5], who showed an iron content ranging from 29.8 to 154.9 μg/g of DW in 74 Andean potato cultivars, processed without distinguishing between flesh and skin tissue. On the other hand, the content of iron in the flesh of our varieties were in the same range as those published by Burgos et al. [17] (9–37 μg/g DW), for several peeled Andean tubers.

A lower content was determined for zinc, with a smaller difference between the two tissues and a mean content of 8.9 μg/g DW. This concentration was lower than that reported by Andre et al. [5] and Lefevre et al. [13], showing mean values of 20.7 μg/g and about 30 μg/g DW, respectively, while Burgos et al. [17] reported similar values in peeled tubers, ranging from 8 μg/g to 20 μg/g DW.

The FAO’s RNI for iron and zinc in adults ranged from 14 to 22 mg/day and from 11 to 14 mg/day, respectively. The high-iron Sangre de Toro genotype contained 12.8 μg/g FW in the flesh and 95.3 μg/g of FW in the skin tissue. As previously described for calcium, we considered a mean value of 20 μg/g FW, according to the flesh-to-skin ratio reported by Jimenez et al. [12]. Considering a mean daily intake for adults of 800 g of potatoes, the Sangre de Toro variety could contribute from 72.8 to 114.4% of the dietary iron intake. These data were in accordance with Andre et al. [5] who showed values even higher, particularly from 10 to 54% in a single potato tuber of about 150 g. Similarly, the consumption of the highest zinc-ranking variety (Puma Makin) containing value of 2.28 μg/g FW could contribute from 13.0 to 16.6% of the dietary zinc intake in adults.

**Figure 2:**
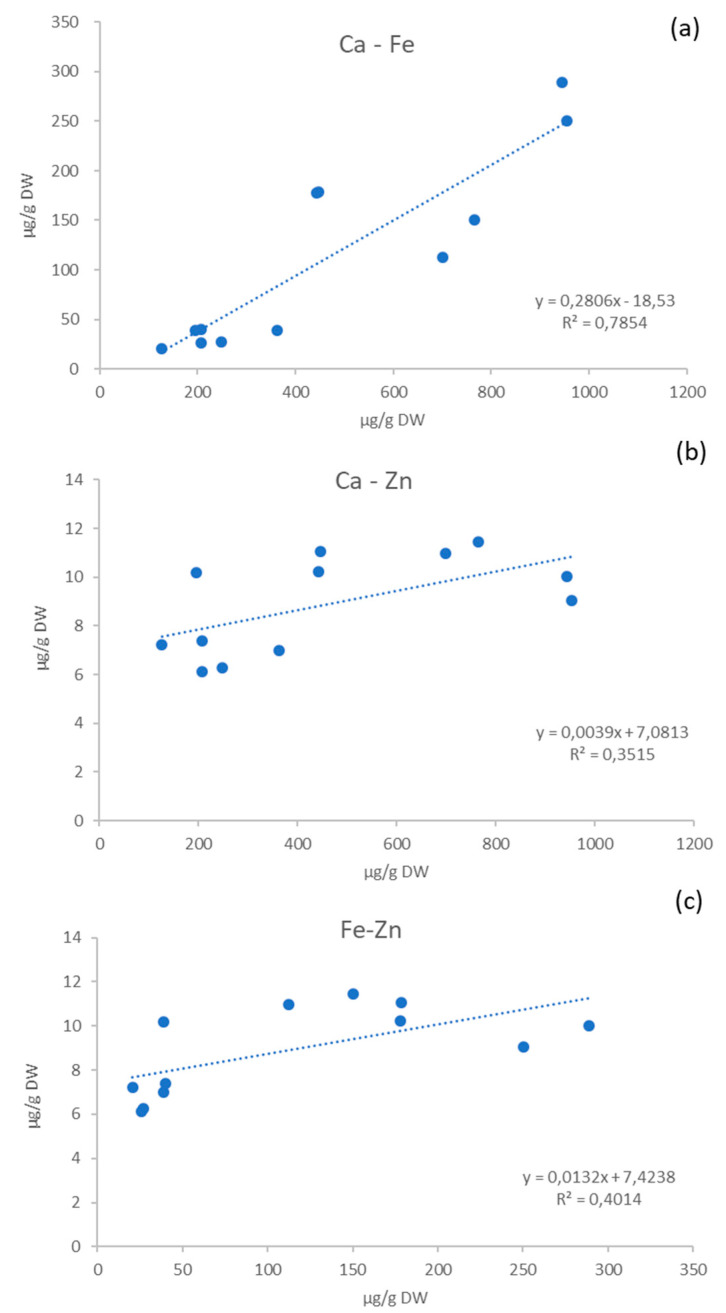
Correlations between calcium and iron (**a**), calcium and zinc (**b**) and iron and zinc (**c**) concentrations (µg/g DW) for the six Andean genotypes.

Calcium, iron, and zinc are among the main elements in potato, and are considered the most important minerals to human health. The correlations between the concentrations of each couple of these elements in the six Andean genotypes are shown in Figure 2. A strong relationship between the calcium and iron content was found (r^2^ = 0.78), in accordance with previous results reported by Andre et al. [5]. Otherwise, only weak correlations between calcium and zinc (r^2^ = 0.35) and iron and zinc (r^2^ = 0.40), were registered.

The concentration of minerals among the six potatoes revealed significant genotypic variation, suggesting genetic differences in the control of this component, as previously reported in other studies [5,15,17]. Moreover, many interactions between plant and soil composition or fertilizer application are likely to affect the concentration of mineral elements in potatoes, as reported in previous studies [18,19,20]. In addition, the climatic conditions can affect the mineral composition of these tubers. Table S1 reports the soil characteristics and climatic conditions of the Paucará district of the Huancavelica region of Peru, where the tubers under study were grown. 

The element uptake depended strongly on the plant and on the growing environment. Soil properties that influence uptake included pH, contents of clay and organic matter, and salinity. Particularly, the uptake of several trace elements in plants tended to be reduced at higher pH and, specifically for potato, significant negative correlations were observed between Cd, Ni, Zn, Mn, Cu, and Al concentration and surface soil pH [21].

In the study of Lombardo et al. [20] a principal component analysis (PCA) showed different accumulations of minerals in cultivars grown in different areas and a genotype effect was observed for all yield and nutritional parameters.

Specific cultivars might provide significant contributions to the intake of several elements, and consumers could significantly increase their mineral intake by consuming the varieties with the higher mineral content, especially with regard to iron and calcium.

### 2.3. Phenolic Compounds and Carotenoids

**Figure 3:**
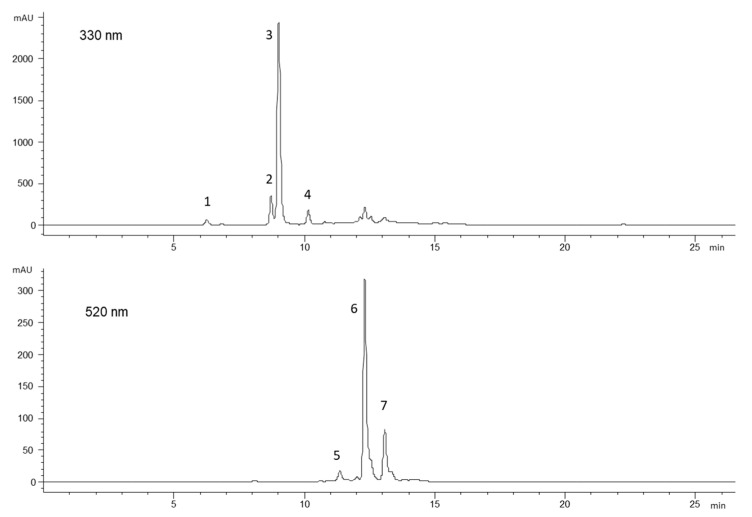
HPLC profiles of the Añil skin extract at 330 nm for phenolic acids and at 520 nm for anthocyanins. (1) 3-*O*-caffeoylquinic acid, (2) cinnamic acid derivative, (3) 5-*O*-caffeoylquinic acid, (4) 4-*O*-caffeoylquinic acid, (5) petunidin 3-*O*-caffeoyl-rutinoside-5-*O*-glucoside, (6) petunidin 3-*O*-coumaroyl-rutinoside-5-*O*-glucoside, and (7) peonidin 3-*O*-coumaroyl-rutinoside-5-*O*-glucoside.

The determination of the main phytochemicals was carried out both on the flesh and skin tissues, to evaluate the distribution of the bioactive compounds in the tuber. The identification of phenolic compounds was performed by HPLC–DAD–MS (Table S2) and the chromatographic profiles showed the presence of the same phenolic pattern in the flesh and skin tissues of each variety. Each sample showed 5-*O*-caffeoylquinic acid as the major phenolic acid and the presence of a predominant anthocyanin, as reported in Figure 3 showed chromatographic profiles at 330 and 520 nm of the Añil skin extract, for example.

The anthocyanins were acylated glycosides of pelargonidin, peonidin, and petunidin, glycosylated with rutinose and glucose that were linked with caffeic, *p*-coumaric, and ferulic acids (Table 4), in accordance with other studies [9,11,22].

**Figure 4:**
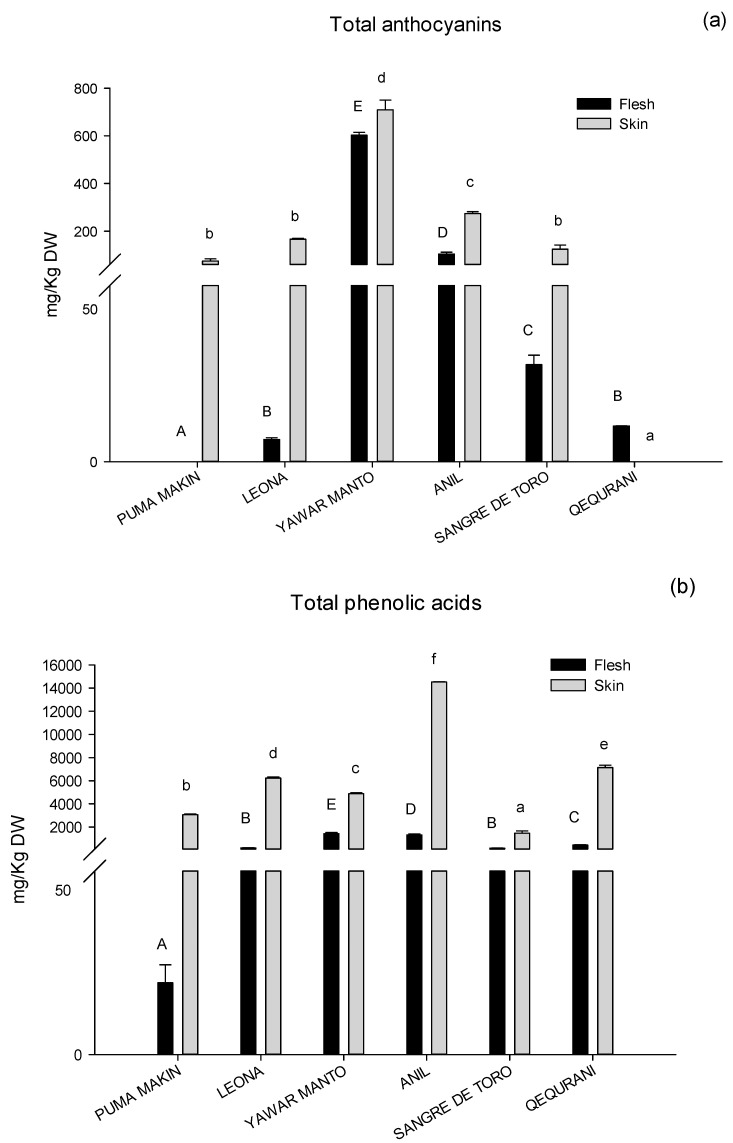
Total anthocyanins (**a**) and total phenolic acids (**b**) in the flesh and skin of the studied tubers. Data are expressed as mg/Kg DW, as mean from triplicates. Different letters indicate significant differences among varieties (*p* < 0.05) for the flesh tissue (uppercase letters) and for the skin tissue (lowercase letters).

The amount of phenolic compounds was calculated according to our previous work [9,23]. The results showed significant qualitative and quantitative differences (*p* < 0.05) among the varieties, and the levels of phenolic compounds were consistently higher in the skin than in the flesh (Figure 4), as largely reported in the literature [9,24,25].

The Puma Makin, Leona, Añil, and Qequrani varieties showed the same pattern of anthocyanins and particularly the presence of petunidin 3-*O-p*-coumaroyl-rutinoside-5-*O*-glucoside and peonidin 3-*O-p*-coumaroyl-rutinoside-5-*O*-glucoside. Nevertheless, significant differences in the content of these pigments were observed among these four varieties—particularly Añil showed the highest content, both in the flesh and in the skin tissues, with values of 80.5 ± 7.2 and 221.3 ± 7.5 mg/Kg dried weight (DW), respectively.

On the other hand, the Sangre de Toro and the Yawar Manto varieties showed the presence of pelargonidin derivatives as typical pigments, and pelargonidin 3-*O*-rutinoside was the most abundant molecule in the two tissues. Significant differences were highlighted in the content of this compound between the two genotypes, and the Yawar Manto variety showed the highest concentration, with similar values for flesh and skin (508.15 ± 11.9 and 574.1 ± 35.2 mg/Kg DW, respectively). The skin extracts of the Yawar Manto variety also showed the presence of two peonidin derivatives, peonidin 3-*O*-rutinoside-5-*O*-glucoside and peonidin 3-*O*-feruloyl-rutinoside-5-*O*-glucoside (Table 4).

The total anthocyanins content in Figure 4a showed significant differences (*p* < 0.05) among the varieties, ranging from 6.3 ± 0.5 to 602.8 ± 12.3 mg/Kg DW for the flesh samples and from 74.3 ± 9.3 to 709.5 ± 40.7 mg/Kg for the skin samples. The Puma Makin variety showed a non-quantifiable amount of anthocyanins in the flesh tissue, while these pigments were undetectable in the skin of the Qequrani variety, in accordance with their flesh and skin colors (Figure 1 and Table 1).

The red Yawar Manto variety registered the highest levels of anthocyanins in both tissues, but also less pronounced differences between flesh and skin, compared to the other varieties. 

With regards to the phenolic acids, the main detected were 3-caffeoylquinic, 5-caffeoylquinic, 4-caffeoylquinic, and caffeic acid (Table 5).

The HPLC–DAD–MS analysis indicated that 5-caffeoylquinic acid was the major phenolic acid in both skin and flesh, for all potato varieties and this was in agreement with other reports [26,27]. Significant differences were observed in its content among the six genotypes; this molecule was more abundant in the Yawar Manto and the Añil varieties for the flesh samples (1046.9 ± 88.7 and 1172,2 ± 78.9 mg/Kg DW, respectively), and in the Añil variety for the skin samples (3893.0 ± 69.5 mg/Kg DW). In addition, the results showed the Puma Makin and the Sangre de Toro as the poorest varieties, both for the flesh and the skin tissues. Following this, caffeic and 4-caffeoylquinic acids were the most abundant phenolic acids in the analyzed extracts. The highest concentration of caffeic acid was in the skin of Qequrani with 809.1 ± 40.4 mg/Kg DW, while the Sangre de Toro and the Yawar Manto genotypes showed the lowest values in the skin (177.2 ± 16.2 and 216.7 ± 5.8 mg/Kg DW, respectively). As for the flesh tissue, the Yawar Manto variety showed the highest concentration with 87.9 ± 0.9 mg/Kg DW. The content of 4-caffeoylquinic acid ranged from 11.9 ± 1.4 to 56.7 ± 4.8 mg/Kg DW for the flesh tissue, while a more pronounced variability was observed for the skin, with values ranging from 105.9 ± 19.1 to 1183.5 ± 14.2 mg/Kg DW. Other minor cinnamic acids were detected in lower amount in potato extracts (Table 5).

Figure 4b compares the values of the total phenolic acids in flesh and skin tissues for the six Andean samples. The quantities varied significantly among the varieties, ranging from 19.5 ± 4.9 to 1412.5 ± 110.7 mg/Kg DW for the flesh tissue and from 1468.1 ± 183.6 to 14533.4 ± 16.4 mg/g DW for the skin tissue. The richest sample was the skin of the Añil variety, while the Yawar Manto genotype showed the highest levels among the flesh samples. The Puma Makin and the Sangre de Toro varieties showed the lowest values both in the flesh and in the skin tissues.

These results were consistent with findings of other authors who reported that variety was one of the most significant factors that influenced the content and the composition of phenolic compounds [6,8,28]. The total phenolic content varied greatly among the six Andean genotypes, in both the flesh and skin tissues. As reported by Andre et al. [6], the potato genotype is the most determining factor that can impact the quantity and the profile of phenolic compounds in potato tubers, although the environmental conditions can also significantly affect the total phenolic content.

Indeed, anthocyanin accumulation in plants is sensitive to environmental conditions—low temperatures enhance anthocyanin accumulation, whereas at high temperatures, the pigment concentration is reduced [29]. Hamouz et al. (2006) reported that different site conditions significantly affect total phenolic content in potato tubers; in two experimental years, the highest phenolic content was determined in the locality characterized by the highest altitude, the lowest average year temperature, and the highest year sum of precipitation, compared to other studied areas [30].

Additionally in the study of Fogelman et al. (2019), it was reported that the growth in hot climates selectively altered potato tuber secondary metabolism—such as the anthocyanins, carotenoids, and glycoalkaloids—changing its nutritive value and the composition of health-promoting components [31].

It can be assumed that the low temperatures and the high altitudes typical of the Andean region where the potatoes under study were grown (Table S1), had a positive impact on the development of these secondary metabolites in the six studied varieties.

The Añil variety, purple fleshed and skinned, was the highest-ranking genotype of phenolic compounds for the skin tissue, while the red Yawar Manto variety was for the flesh one.

The different patterns of anthocyanins defined the color of the variety; the red genotypes (Sangre de Toro and Yawar Manto) contained predominantly pelargonidin and peonidin derivatives, while the purple varieties (Puma Makin, Leona, Añil and Qequrani) had petunidin and peonidin as major anthocyanidins, in accordance with the literature [7,9,11,23,25]. This class of pigments is recognized as the phenolic fingerprint and can be used for varietal recognition in red-violet potatoes, as reported by Ieri et al. [9], and also in other crops like *Vitis vinifera* L. red grapes [32].

Our data did not show a correlation between the levels of total phenols in the flesh and in the skin; the varieties with the highest levels in the flesh did not always correspond to the highest levels in the skin. These results differed from those reported by Valcarcel et al. [24], which showed a significant correlation between the two tissues, but were in agreement with our previous data [9], not showing an absolute correlation in the content of phenolic acids between flesh and skin tissues.

With regards to carotenoids, the method reported by Les et al. [33] was used to extract these pigments from the flesh. The organic extracts of all Andean varieties did not show any presence of carotenoids in their chromatographic profiles at 430 nm, even though the analysis of more concentrated samples (five time higher) was also performed to improve the sensibility of the method. These molecules were below the limit of detection of the applied method, confirming the absence of carotenoids in the flesh of these genotypes.

Burgos et al. [34] highlighted the significant genetic diversity existing for total and individual carotenoids in *S. phureja* potatoes. Similarly, Andre at al. [5] reported a large variation in the total carotenoid content (2.8-36.2 µg/g DW) among 74 potato genotypes of different taxonomic groups of the *S. tuberosum* species, highlighting the highest variability for the Andigenum group, as well as a strong correlation between carotenoid concentration and yellowness. More recently, Valcarcel et al. [24], based on the analysis of 60 potatoes (*S. tuberosum* L.), reported that variety had a significant effect on the carotenoid content, showing levels in the flesh below the limit of detection for white- and blue-fleshed varieties, and only a maximum amount close to 1.1 mg/Kg DW, for the other samples.

## 3. Materials and Methods

### 3.1. Plant Materials and Sample Preparation

Six varieties of Andean potatoes (Puma Makin, Leona, Yawar Manto, Añil, Sangre de Toro, and Qequrani) of different colors and species (*Solanum andigenum, Solanum stenotomum*, and *Solanum goniocalyx*) were studied. The crops were grown in fields located in the district of Paucará, in the province of Acobamba (altitude 3500–4100 m above sea level) in the Huancavelica region of Peru and were purchased from a local producer (Paccho Molinos Farlands) who classified them according to the International Potato Center (CIP) guideline. 

The main morphological characteristics of the tubers are summarized in Table 1. The analyses were conducted on composite samples of each variety. Potatoes were washed with water and peeled; skin and flesh were stored at −22 °C, until the freeze-dry process. The dried flesh and skin of potatoes were treated with liquid nitrogen, then ground to obtain a homogenous powder.

### 3.2. Proximate Analysis

The proximate composition (moisture, protein, fat, carbohydrates, and ash) of the powdered samples was carried out according to the AOAC official methods (2012). The moisture content was determined by oven drying the sample to a constant weight (AOAC 920.151). Total protein content was determined by the Kjeldahl method with a nitrogen-to-protein conversion factor of 6.25 (AOAC 920.152). The ash content was determined by incineration at 600 °C, in a muffle furnace (AOAC 940.26 (A)). The crude fat content was determined by the Soxhlet extraction (AOAC 930.09). Total carbohydrate content was calculated as a difference, using the following formula—Carbohydrates (%) = 100 – (% crude protein + % total ash + % crude fat). The total energy content was expressed in calories, using the conversion factors 4.0 for crude proteins, 9.0 for crude fat, and 4.0 for carbohydrates.

### 3.3. Determination of the Mineral Content

Macro and microelement analysis was performed using 0.5 mg of dried sample digested with 10 mL of HNO_3_ (67% *v*/*v*) in Teflon reaction vessels, to perform the mineralization in a microwave oven (Mars 5, CEM Corp., Matthews, NC, USA), using the program 1600 W, 100% power, at 200 °C for 20 min. At the end of the mineralization, the final volume of 25 mL was reached by adding ultra-pure water.

The concentrations of Ca, K, Mg, Na, P, Cu, Fe, Mn, and Zn were determined using an inductively coupled argon plasma optical emission spectrometer (ICP–OES iCAP series 7000 Plus Thermo Scientific). A standard method for the 24 different elements was applied, using the Qtegra^TM^ Intelligent Scientific Data Solution^TM^ (ISDS) and the wavelengths selected were 315.8 nm for Ca, 766.4 nm for K, 285.2 nm for Mg and Na, 178.7 nm for P, 324.7 nm for Cu, 239.1 for Fe, 259.3 nm for Mn, and 202.5 nm for Zn quantification. The calibration was performed with several dilutions of the multi-element standard Astasol^®^-Mix (ANALYTIKA^®^, spol. s.r.o., Prague, Czech Republic) in 1% HNO_3_ (*v*/*v*).

### 3.4. Extraction of Phenolic Compounds

The homogeneous dry powdered material (1 g) was extracted twice with 30 mL of 70% EtOH, adjusted to pH 2.0 by HCOOH, as already described [9]; the supernatant was filtered, dried under vacuum, and re-dissolved with 5–10 mL of the following mixture—acidic water/acetonitrile/methanol 8:1:1 *v*/*v*/*v*, with 2.0 as final pH. The hydro-alcoholic solutions obtained after centrifugation were analyzed by HPLC–DAD–MS.

### 3.5. Extraction of Carotenoids

Carotenoids were extracted according to the method reported by Les et al. (2017) [33]. In brief, 1 g of freeze-dried powder was extracted twice with 35 mL of the mixture ethanol/hexane 55:45 *v*/*v*, under magnetic stirring for 20 min, and then filtered. The extract was dried under vacuum (–0.1 MPa, and 30 °C) by Rotavapor^®^ R-100 (Büchi); the dry extract was re-dissolved in an exact volume of acetone, and then analyzed by HPLC–DAD. All manipulations were carried out under subdued artificial light conditions.

### 3.6. HPLC–DAD–MS Analysis

The analyses were performed using a HP1200 liquid chromatograph equipped with a DAD detector (Agilent Technologies, Palo Alto, CA, USA).

The identification of the phenolic compounds was performed by HPLC–PDA–MS on a Waters (Manchester, UK) system, composed by an Alliance 2695 HPLC, a PDA 2996, and a Quattro micro triple quadrupole mass spectrometer, equipped with a Z-spray ESI interface. The separation of phenolic compounds was performed using a Synergi Max RP 80 A column (150 × 3 mm i.d.; 4 μm particle size, Phenomenex, Castelmaggiore, Bologna, Italy). The mobile phases were: (A) acidified water (pH 2.0) and (B) acetonitrile. The following multistep linear gradient was applied—from 95% to 78% A in 8 min, 4 min to reach 74% A, then 13 min to arrive at 65% A, and finally 3 min to reach 100% B, with a final plateau of 4 min. The total time of analysis was 32 min, flow rate was 0.4 mL/min, and oven temperature was 26 ± 0.5 °C, as described in our previous study [9]. The injection volume was 10 µL. The eluent from the column was split to the PAD detector and to the mass spectrometer in a 2:1 (*v*/*v*) ratio. The UV spectra were recorded from 250 to 600 nm. The mass spectrometer was operated in negative and positive ion mode, to detect phenolic acids and anthocyanins, respectively. Mass spectra were recorded in scan mode from 100 *m*/*z* to 1000 *m*/*z* at 1 s/scan time. MS parameters were set up as follows—source block and desolvation gas temperature at 130 and 380 °C, respectively, capillary voltage was 3.2 kV, cone gas and desolvation gas flow was 20 and 350 L/h, respectively. The MassLynx software was used for data acquisition and processing. 

The analyses of carotenoids were carried out according to Les et al. [33], using a Luna RP 18 column (150 × 3 mm, 5 μm) from Phenomenex. The mobile phases were 0.1% formic acid in water (A) and acetone (B); a multistep linear solvent gradient was applied—in 0 to 20 min from 80% B to 100% B, with a final plateau of 5 min; equilibration time was 10 min, flow rate was 0.4 mL min^−1^, and oven temperature was 26 °C.

### 3.7. Quantitative Determination of Phenolic Compounds

Chromatograms were registered at 330 and 520 nm for phenolic acids and anthocyanins, respectively. These compounds were identified by comparing their retention times, UV–Vis, and MS spectra, with those of the respective standard when possible, or with our previous published data [9,11]. A six-point calibration curve of chlorogenic acid (Extrasynthèse, Genay, France; purity 99%) at 330 nm (r^2^ = 0.999) was used to evaluate the phenolic acids, while the anthocyanin content was determined using a six-point calibration curve of malvin chloride (MW 691, Extrasynthèse, purity 95%) at 520 nm (r^2^ = 0.999).

### 3.8. Statistical Analysis

Analyses were carried out in triplicates and the results were expressed as mean ± standard deviation. Analysis of variance and F-test (*p* < 0.05) were performed using Microsoft Excel statistical software to evaluate statistical significance. Fisher’s LSD test was applied to compare the means using the software DSAASTAT v. 1.1.

## 4. Conclusions

This study helps to increase the knowledge on the quantity and quality of nutrients and functional compounds in native Andean potatoes. The six Andean varieties included in this study showed a great variability in minerals, like macronutrients and micronutrients, and in phenolic content. Mineral concentrations in the tubers revealed significant genotypic variation, highlighting that some of these genotypes could be used in the diet as a significant source of essential nutrients like iron. Particularly, the Sangre de Toro variety showed the highest content in calcium and iron in the fresh tubers, both in the flesh and in the skin samples.

Analogously, the genotypic biodiversity also contributed to the wide variability observed for the phenolic content, with levels of phenolic compounds being consistently higher in the skin than in the flesh. The phenolic acids were the most abundant class in all samples and the Añil variety resulted in the richest tuber for the skin tissue, while the Yawar Manto genotype showed the highest levels among the flesh samples.

The color of the variety turned out to be associated to a different pattern of anthocyanins, and the red Yawar Manto genotype showed the highest levels of total anthocyanins in both tissues. 

As for the carotenoids content, the flesh extracts of these Andean varieties did not show the presence of these pigments.

By providing data to allow the diversification of the production, it is possible to contribute to the conservation of biodiversity. Knowledge on variations within the potato germplasm offers the possibility of choosing high-ranking varieties, in terms of minerals or phenolic compounds, in order to improve the food and nutritional security of local populations, through a more balanced diet and, at the same time, avoiding the loss of biodiversity.

## Figures and Tables

**Table 1:** Main morphological characteristics of the studied accessions of Andean potatoes.

	Species	Tuber Form	Skin Color	Flesh Color		Predominant Bud Color	Flower Color	Stem Color
				Main	Secondary			
Puma Makin	*S. tuberosum andigenum*	Oblong fingered	Blackish	Pale yellow	Violet	Purple	Pale purple	Purple
Leona	*S. tuberosum andigenum*	Round	Blackish	Purple	Yellow (few spots)	Purple	Pale violet	Purple
Yawar Manto	*S. tuberosum andigenum*	Elongate	Blackish	Dark red	-	Red	White with red dots	Red
Añil	*S. tuberosum andigenum*	Oblong	Blackish	Dark purple	-	Purple with white tip	Purple with white dots	Black
Sangre De Toro	*S. goniocalyx*	Round	Brown	Red	Yellow (few spots)	Yellow with red tip	Intense pink	Red
Qequrani	*S. stenotomum*	Oblong	Pale brown	Pale yellow	Violet	Purple	White	Green with spots

**Table 2:** Proximate composition of the flesh and skin of the six varieties of Andean potatoes. The values of relative standard deviation were below 7%.

	Puma Makin		Leona		Yawar Manto		Añil		Sangre De Toro		Qequrani	
	**Flesh**	**Skin**	**Flesh**	**Skin**	**Flesh**	**Skin**	**Flesh**	**Skin**	**Flesh**	**Skin**	**Flesh**	**Skin**
Whole tuber Kcal/100g	129.8	106.4	117.4	91.4	90.3	99.4	98.1	131.0	131.1	150.4	150.6	105.3
% Kcal from carbohydrates	88.1	80.1	88.3	80.1	79.7	86.9	84.4	90.4	84.5	88.69	90.6	86.6
**Data Expressed as g/100 g**												
Moisture	66.6	72.7	69.8	76.8	76.5	74.4	73.9	66.2	66.8	61.9	61.4	72.4
Carbohydrates	28.6	21.3	25.6	18.3	18.0	21.6	20.7	29.7	27.7	33.3	34.1	22.8
Fats	0.2	0.4	0.2	0.6	0.3	0.2	0.1	0.2	0.7	0.4	0.2	0.1
Proteins	3.4	4.4	3	3.2	3.9	3.4	3.6	2.7	1.3	3.4	3.1	3.3
Ash	1.2	1.2	1.1	1.1	1.3	1.0	1.7	1.3	1.3	1.0	1.2	1.4

**Table 3:** Mineral content in the tubers expressed as μg/g dry weight (DW). Data are expressed as mean ± standard deviation. Different letters indicate significant differences among varieties, for each compound (*p* < 0.05) for the flesh tissue (uppercase letters) and for the skin tissue (lowercase letters).

	Puma Makin		Leona		Yawar Manto	
	**Flesh**	**Skin**	**Flesh**	**Skin**	**Flesh**	**Skin**
Ca	207.6 ± 18.4 ^B^	766.1 ± 19.5 ^c^	206.5 ± 10.7 ^B^	699.9 ± 14.9 ^b^	195.3 ± 12.1 ^B^	446.7 ± 22.4 ^a^
K	7272.9 ± 129.7 ^A^	13071.8 ± 102.8 ^b^	8040.3 ± 22.1 ^C^	14537.4 ± 230.3 ^d^	13059.9 ± 36.5 ^F^	17388.6 ± 211.1 ^e^
Mg	351.6 ± 14.8 ^B^	525.9 ± 12.0 ^b^	374.2 ± 7.2 ^C^	627.4 ± 19.9 ^c^	591.4 ± 8.7 ^F^	692.4 ± 14.9 ^d^
Na	19.9 ± 8.3 ^A^	33.1 ± 1.8 ^a^	17.5 ± 3.6 ^A^	31.1 ± 2.4 ^a^	31.1 ± 0.6 ^BC^	46.1 ± 3.0 ^b^
P	1140.7 ± 9.3 ^C^	1835.7 ± 6.3 ^c^	1181.6 ± 26.5 ^D^	2124.9 ± 24.9 ^e^	1252.1 ± 21.1 ^E^	1866.4 ± 33.2 ^c^
Cu	1.5 ± 0.0 ^B^	3.6 ± 0.1 ^d^	1.9 ± 0.0 ^C^	4.0 ± 0.0 ^e^	2.1 ± 0.0 ^C^	3.6 ± 0.0 ^d^
Fe	39.9 ± 6.5 ^C^	150.2 ± 10.7 ^b^	26.1 ± 1.2 ^AB^	112.2 ± 6.2 ^a^	38.9 ± 3.6 ^C^	178.4 ± 27.0 ^c^
Mn	5.6 ± 0.2 ^B^	7.6 ± 0.2 ^a^	5.5 ± 0.1 ^C^	7.9 ± 0.2 ^a^	9.8 ± 0.1 ^E^	18.8 ± 0.3 ^d^
Zn	7.4 ± 0.9 ^B^	11.4 ± 0.5 ^c^	6.1 ± 0.8 ^A^	10.9 ± 0.5 ^c^	10.2 ± 0.6 ^C^	11.0 ± 0.5 ^c^
	**Anil**		**Sangre De Toro**		**Qequrani**	
	**Flesh**	**Skin**	**Flesh**	**Skin**	**Flesh**	**Skin**
Ca	126.2 ± 13.4 ^A^	443.4 ± 5.1 ^a^	361.9 ± 7.8 ^D^	953.5 ± 10.1 ^d^	248.4 ± 15.3 ^C^	943.6 ± 7.9 ^d^
K	10426.4 ± 88.7 ^E^	17124.2 ± 281.9 ^e^	7542.7 ± 60.7 ^B^	12418.0 ± 209.6 ^a^	8326.9 ± 80.5 ^D^	13907.8 ± 211.5 ^c^
Mg	528.0 ± 9.6 ^E^	833.1 ± 12.8 ^e^	312.1 ± 3.8 ^A^	414.2 ± 13.7 ^a^	446.5 ± 6.6 ^D^	516.2 ± 11.8 ^b^
Na	18.0 ± 0.2 ^A^	45.4 ± 3.8 ^b^	37.9 ± 3.3 ^C^	35.7 ± 6.5 ^a^	22.3 ± 9.9 ^AB^	39.9 ± 9.8 ^ab^
P	1074.1 ± 6.2 ^B^	1973.6 ± 49.5 ^d^	924.5 ± 11.4 ^A^	1464.4 ± 26.8 ^a^	1113.7 ± 21.5 ^C^	1669.1 ± 19.3 ^b^
Cu	1.0 ± 0.0 ^A^	2.3 ± 0.0 ^a^	1.5 ± 0.3 ^B^	2.8 ± 0.0 ^b^	1.2 ± 0.0 ^A^	2.9 ± 0.0 ^c^
Fe	20.5 ± 1.2 ^A^	178.1 ± 7.9 ^c^	38.7 ± 0.5 ^C^	250.2 ± 12.1 ^d^	26.8 ± 1.4 ^B^	288.8 ± 5.3 ^e^
Mn	6.7 ± 0.0 ^D^	18.8 ± 0.4 ^d^	4.7 ± 0.0 ^A^	11.4 ± 0.9 ^b^	5.1 ± 0.0 ^B^	14.4 ± 0.2 ^c^
Zn	7.2 ± 0.3 ^AB^	10.2 ± 0.1 ^b^	7.0 ± 0.5 ^AB^	9.0 ± 0.2 ^a^	6.3 ± 0.9 ^AB^	10.0 ± 0.3 ^b^

**Table 4:** Anthocyanins in the flesh and skin of the different tubers expressed as mg/Kg DW. Data are expressed as mean ± standard deviation. Different letters indicate significant differences among varieties for each compound (*p* < 0.05) for the flesh tissue (uppercase letters) and for the skin tissue (lowercase letters).

Variety	Puma Makin						Leona					
	**Flesh**			**Skin**			**Flesh**			**Skin**		
**Compound**	**mg/Kg**	**SD**		**mg/Kg**	**SD**		**mg/Kg**	**SD**		**mg/Kg**	**SD**	
pet 3-*O*-caf-rut-5-*O*-glu	nd	-	A	nd	-	a	nd	-	A	nd	-	a
pet 3-*O-p*-coum-rut-5-*O*-glu	nd	-	A	43.6	7.1	b	5.35	0.5	B	121.4	3.6	c
pet 3-*O*-ferul-rut-5-*O*-glu	nd	-	A	nd	-	a	nd	-	A	nd	-	a
peo 3-*O*-rut-5-*O*-glu	nd	-	A	nd	-	a	nd	-	A	nd	-	a
peo 3-*O*-*p*-coum-rut-5-*O*-glu	nd	-	A	30.7	2.3	b	0,97	0.0	B	45.25	1.3	c
peo 3-*O*-ferul-rut-5-*O*-glu	nd	-	A	nd	-	a	nd	-	A	nd	-	a
pel 3-*O*-rut-5-*O*-glu	nd	-	A	nd	-	a	nd	-	A	nd	-	a
pel 3-*O*-rut	nd	-	A	nd	-	a	nd	-	A	nd	-	a
pel 3-*O*-*p*-coum-rut-5-*O*-glu	nd	-	A	nd	-	a	nd	-	A	nd	-	a
pel 3-*O*-ferul-rut	nd	-	A	nd	-	a	nd	-	A	nd	-	a
pel 3-*O*-cis*-p*-coum-rut-5-*O*-glu	nd	-	A	nd	-	a	nd	-	A	nd	-	a
**Variety**	**Yawar Manto**						**Anil**					
	**Flesh**			**Skin**			**Flesh**			**Skin**		
**Compound**	**mg/Kg**	**SD**		**mg/Kg**	**SD**		**mg/Kg**	**SD**		**mg/Kg**	**SD**	
pet 3-*O*-caf-rut-5-*O*-glu	nd	-	A	nd	-	a	4.1	0.1	B	nd	-	a
pet 3-*O-p*-coum-rut-5-*O*-glu	nd	-	A	nd	-	a	80.5	7.2	C	221.3	7.5	d
pet 3-*O*-ferul-rut-5-*O*-glu	nd	-	A	nd	-	a	6.1	0.6	B	nd	-	a
peo 3-*O*-rut-5-*O*-glu	nd	-	A	15.8	1.6	b	nd	-	A	nd	-	a
peo 3-*O*-*p*-coum-rut-5-*O*-glu	nd	-	A	nd	-	a	13.3	0.8	D	52.6	1.7	d
peo 3-*O*-ferul-rut-5-*O*-glu	nd	-	A	66.6	0.4	b	nd	-	A	nd	-	a
pel 3-*O*-rut-5-*O*-glu	15.0	0.4	B	52.9	4.2	b	nd	-	A	nd	-	a
pel 3-*O*-rut	508.2	11.9	C	574.1	35.2	c	nd	-	A	nd	-	a
pel 3-*O*-*p*-coum-rut-5-*O*-glu	nd	-	A	nd	-	a	nd	-	A	nd	-	a
pel 3-*O*-ferul-rut	54.3	1.1	C	nd	-	a	nd	-	A	nd	-	a
pel 3-*O*-cis*-p*-coum-rut-5-*O*-glu	25.4	0.1	B	nd	-	a	nd	-	A	nd	-	a
**Variety**	**Sangre De Toro**						**Qequrani**					
	**Flesh**			**Skin**			**Flesh**			**Skin**		
**Compound**	**mg/Kg**	**SD**		**mg/Kg**	**SD**		**mg/Kg**	**SD**		**mg/Kg**	**SD**	
pet 3-*O*-caf-rut-5-*O*-glu	nd	-	A	nd	-	a	nd	-	A	nd	-	a
pet 3-*O-p*-coum-rut-5-*O*-glu	nd	-	A	nd	-	a	6.8	0.0	B	nd	-	a
pet 3-*O*-ferul-rut-5-*O*-glu	nd	-	A	nd	-	a	nd	-	A	nd	-	a
peo 3-*O*-rut-5-*O*-glu	nd	-	A	nd	-	a	nd	-	A	nd	-	a
peo 3-*O*-*p*-coum-rut-5-*O*-glu	nd	-	A	nd	-	a	3.4	0.0	C	nd	-	a
peo 3-*O*-ferul-rut-5-*O*-glu	nd	-	A	nd	-	a	nd	-	A	nd	-	a
pel 3-*O*-rut-5-*O*-glu	nd	-	A	nd	-	a	nd	-	A	nd	-	a
pel 3-*O*-rut	23.5	2.9	B	113.5	17.4	b	nd	-	A	nd	-	a
pel 3-*O*-*p*-coum-rut-5-*O*-glu	1.1	0.0	B	3.9	0.2	b	nd	-	A	nd	-	a
pel 3-*O*-ferul-rut	2.9	0.2	B	6.8	0.4	b	nd	-	A	nd	-	a
pel 3-*O*-cis*-p*-coum-rut-5-*O*-glu	nd	-	A	nd	-	a	nd	-	A	nd	-	a

pet, petunidin; peo, peonidin; pel, pelargonidin; caf, caffeoyl; ferul, feruloyl; *p*-coum, *p*-coumaroyl; rut, rutinoside; glu, glucoside; and nd, not detected.

**Table 5:** Phenolic acids in the flesh and skin of the different tubers expressed as mg/Kg DW. Data are expressed as mean ± standard deviation. Different letters indicate significant differences among varieties for each compound (*p* < 0.05) for the flesh tissue (uppercase letters) and for the skin tissue (lowercase letters).

Variety	Puma Makin						Leona					
	**Flesh**			**Skin**			**Flesh**			**Skin**		
**Compound**	**mg/Kg**	**SD**		**mg/Kg**	**SD**		**mg/Kg**	**SD**		**mg/Kg**	**SD**	
cinnamic acid	nd	-	A	nd	-	a	nd	-	A	nd	-	a
3-caffeoyquinic acid	nd	-	A	48.7	0.8	b	nd	-	A	117.2	3.4	d
5-caffeoyquinic acid	7.6	4.1	A	2221.2	26.3	b	156.4	17.7	AB	4807.8	79.8	d
cinnamic acid	nd	-	A	nd	-	a	nd	-	A	nd	-	a
4-caffeoyquinic acid	11.9	1.4	A	210.0	2.2	b	12.0	0.9	A	865.7	10.0	e
caffeic acid	nd	-	A	500.0	33.3	c	19.7	1.3	B	299.6	14.9	b
cinnamic acid	nd	-	A	27.3	4.7	b	5.8	0.4	B	140.8	2.6	d
cinnamic acid	nd	-	A	28.6	0.6	c	nd	-	A	nd	-	a
cinnamic acid	nd	-	A	25.6	1.0	b	nd	-	A	nd	-	a
**Variety**	**Yawar Manto**						**Anil**					
	**Flesh**			**Skin**			**Flesh**			**Skin**		
**Compound**	**mg/Kg**	**SD**		**mg/Kg**	**SD**		**mg/Kg**	**SD**		**mg/Kg**	**SD**	
cinnamic acid	54.2	2.2	B	nd	-	a	nd	-	A	nd	-	a
3-caffeoyquinic acid	58.7	4.8	B	76.0	0.4	c	nd	-	A	248.8	4.9	f
5-caffeoyquinic acid	1046.9	88.7	C	3893.0	69.5	c	1172.1	78.9	C	12173.3	37.3	f
cinnamic acid	27.0	3.1	C	nd	-	a	nd	-	A	nd	-	a
4-caffeoyquinic acid	56.7	4.8	D	595.8	8.1	d	53.8	4.4	C	1183.4	14.2	f
caffeic acid	87.9	0.9	C	216.7	5.8	a	23.2	1.8	B	579.2	56.1	d
cinnamic acid	25.4	2.6	D	48.2	6.9	c	59.4	4.5	E	348.5	17.7	e
cinnamic acid	35.1	2.5	D	49.9	1.6	d	nd	-	A	nd	-	a
cinnamic acid	20.4	1.1	C	nd	-	a	nd	-	A	nd	-	a
**Variety**	**Sangre De Toro**						**Qequrani**					
	**Flesh**			**Skin**			**Flesh**			**Skin**		
**Compound**	**mg/Kg**	**SD**		**mg/Kg**	**SD**		**mg/Kg**	**SD**		**mg/Kg**	**SD**	
cinnamic acid	nd	-	A	nd	-	a	nd	-	A	nd	-	a
3-caffeoyquinic acid	nd	-	A	nd	-	a	nd	-	A	136.0	16.8	e
5-caffeoyquinic acid	97.2	5.7	A	1114.3	166.3	a	363.7	19.3	B	5681.9	70.6	e
cinnamic acid	12.2	0.3	B	32.5	1.2	b	nd	-	A	nd	-	a
4-caffeoyquinic acid	13.2	0.9	A	105.9	19.1	a	21.7	0.6	B	518.3	83.8	c
caffeic acid	17.3	0.9	B	177.1	16.2	a	34.5	3.3	B	809.1	40.4	e
cinnamic acid	10.5	0.6	C	30.0	2.7	b	5.5	0.0	B	nd	-	a
cinnamic acid	6.0	0.4	B	8.2	1.5	b	10.1	0.2	C	nd	-	a
cinnamic acid	14.9	0.3	B	nd	-	a	nd	-	A	nd	-	a

nd, not detected.

## Supplementary Materials

The following are available online, Table S1: Soil characteristics and climatic conditions of the Paucará district (Acobamba province of Huancavelica region of Peru). The values are taken from bibliographical sources [35,36,37], Table S2: MS data obtained in the positive and negative ion mode of the anthocyanins and the phenolic acids found in the potato extracts.
