# Supplementary material for: A Study on the Biodiversity of Pigmented Andean Potatoes: Nutritional Profile and Phenolic Composition
Source: Molecules. 2020 Jul 10;25(14):3169. doi: 10.3390/molecules25143169 (PMC7397087; doi:10.3390/molecules25143169)
Supplement: Supplementary file 1 [file molecules-25-03169-s001.pdf]

Supplemetary Materials

Table 1. Soil characteristics and climatic conditions of the Paucará district (Acobamba province of Huancavelica region of Peru). The values are taken from bibliographical sources [35–37].

	Values
**Annual Relative Umidity**	43% - 65%
**Altitude**	3800 asl
**Annual rainfall**	398-800 mm
**Mean temperature**	12-14 °C
**N total (%)**	0.24 (high)
**P (ppm)**	4.30 (low)
**K (ppm)**	115 (medium)
**pH**	4.6
**Sand (%)**	32.8
**Slime (%)**	43.6
**Clay (%)**	23.6

Table S2. MS data obtained in the positive and negative ion mode of the anthocyanins and the phenolic acids found in the potato extracts.

**Anthocyanins**	**Rt (min)**	**Molecular ion (m/z)**	**Fragment ions and adducts (m/z)**
pet 3-*O*-caf-rut-5-*O*-glu	11.2	949	317, 479, 787
pet 3-*O*-*p*-coum-rut-5-*O*-glu	12.5	933	317, 479, 771, 787
pet 3-*O*-ferul-rut-5-*O*-glu	12.6	963	317, 479, 787, 801
peo 3-*O*-rut-5-*O*-glu	9.1	771	301, 463, 609
peo 3-*O*-*p*-coum-rut-5-*O*-glu	13.1	917	301, 463, 755, 771
peo 3-*O*-ferul-rut-5-*O*-glu	13.3	947	301, 463, 771,785
pel 3-*O*-rut-5-*O*-glu	8.4	741	271, 433, 579
pel 3-*O*-rut	10.6	579	271
pel 3-*O*-*p*-coum-rut-5-*O*-glu	8.0	887	271, 433, 725, 741
pel 3-*O*-ferul-rut	11.9	755	271
pel 3-*O*-cis-*p*-coum-rut-5-*O*-glu	13.0	887	271, 433, 725

**Phenolic acids**	**Rt (min)**	**Molecular ion (m/z)**	**Fragment ions and adducts (m/z)**
cinnamic acid	5.5	315	669
3-caffeoyquinic acid	6.3	353	375, 191, 179
5-caffeoyquinic acid	9.1	353	729, 375, 191
cinnamic acid	9.5	443	267

4-caffeoyquinic acid	10.1	353	383, 191
caffeic acid	10.3	179	-
cinnamic acid	10.7	-	431
cinnamic acid	10.9	443	267
cinnamic acid	11.1	-	471
